# Will the “Environmental Fees to Taxes” Affect Firm Charitable Giving?

**DOI:** 10.3390/ijerph192315667

**Published:** 2022-11-25

**Authors:** Feng Niu, Jiayi Wang, Wunhong Su

**Affiliations:** School of Accounting, Hangzhou Dianzi University, Hangzhou 310018, China

**Keywords:** tax burden criteria, firm heterogeneity, differences-in-differences

## Abstract

This study examines the impact of the 2018 “environmental protection fee to tax” policy on the charitable giving of polluting firms between 2015 and 2019 using a differences-in-differences method. This study found that implementing the “environmental protection fee to tax” policy reduced the level of charitable giving by polluters. The decline in charitable-giving levels was more pronounced among firms classified as heavy polluters, firms from the East of China, and non-state firms. The results suggest that the “environmental protection fee to tax” policy cannot only encourage firms to become more environmentally conscious but can also be used to determine the motivations behind firm charitable donations. The policy of changing environmental protection fees to taxes needs to be effectively implemented in China and strengthen the implementation effect.This study enriches the literature on environmental policies and firm charitable giving and provides empirical evidence on the economic consequences of the “environmental protection fee to tax” policy. It can also help polluters and regulators to understand the “environmental protection fee better to tax” policy and help the government to improve the external systems that regulate and guide corporate social responsibility.

## 1. Introduction

According to the 2019 China Charitable Donation Report released by the China Charity Federation at the 8th China Charity Exhibition, China accepted a total of CNY 170.144 billion in domestic and foreign donations in 2019, of which firm donations amounted to CNY 93.147 billion, accounting for about 54.75%. It is evident that China’s firms are the backbone of social charity donations and have made important contributions to social initiatives such as disaster relief, schooling, and poverty alleviation. However, many “disharmonious” hypocritical practices related to charitable donations still occur. For example, it is not uncommon for firms to use charitable donations to seek rent from the government for profit [1]. Some firms even use charitable donations to cover negative news [2]. For example, Jinpu Cement Firm, a major polluter, donated 100 tons of cement to repair a road in China. Due to their strong negative environmental externalities, polluting firms are incentivized to use charitable donations to seek a more relaxed regulatory environment. As a result, they can evade their environmental responsibilities in the face of local environmental authorities’ management [3,4,5,6].

In recent years, China’s environmental protection mechanism has been constantly updated and improved, with a fast pace of reform, strong efforts, and strict accountability, positioning environmental protection as a new era towards ecological civilization. For example, the Chinese State Council proposed the Interim Measures for the Collection of Sewage Charges in 1982, arousing concern about environmental issues with polluting firms and the public. Although implementing the scheme raised a lot of funds for environmental protection, there are many issues with sewage charge collection, such as irregular charging procedures, corruption in environmental protection sectors, and narrow coverage of levy recipients, which are gradually being exposed to the public and whose seriousness is becoming increasingly evident. Accordingly, the “environmental protection fee to tax” policy was officially implemented in early 2018.

Compared to the administrative charges of the environmental protection sector during the emission fee period, the environmental protection tax is charged by the taxation sector, which specializes in collecting taxes and fees, and uses a standardized and independent tax law as the basis for the collection. Therefore, adjusting the charging method systematically reduces the possibility of rent seeking by paying fees, improves the rigidity of tax enforcement, and strengthens the responsibility of firms to reduce pollution [7]. In addition, the previous regulation for firms that refused to pay sewage charges or concealed omissions was a fine of up to 33 times the charge amount or an order to suspend rectification and other forms of punishment. Therefore, the previous deterrent effect on polluting firms was inadequate. Under the “environmental protection fee to tax” policy, the regulation ensures that a firm that is found to evade taxes can face a fine of up to 5 times the amount of bear criminal liability, greatly increasing the cost to polluting firms.

An important issue is whether the charitable donations made by polluting firms are made in a more regulated institutional environment after implementing the “environmental fee to tax” policy. Do polluting firms’ charitable donations start with good intentions? Existing studies on the “environmental fee to tax” policy mainly focus on firm performance [8], technological innovation [9,10,11,12], and information disclosure [13]. Few studies discuss the relationship between the “environmental protection fee to tax” policy and the charitable donations of polluting firms. This study considers the implementation of the “environmental protection fee to tax” policy in January 2018 as an exogenous event. It utilizes all listed firms with primary industry classifications B, C, and D in Shanghai and Shenzhen with A-shares from 2015 to 2019. These firms comprised the initial sample used to test the association between the “environmental protection fee to tax” policy and the charitable donations of polluting firms, which was conducted using the differences-in-differences (DID) method. The results show that charitable donations by polluting firms dropped significantly after the “environmental protection fee to tax” policy was implemented.

Furthermore, the charitable donations made by heavy-polluting firms declined significantly relative to the light- and medium-polluting firms. Relative to state-owned enterprises (SOEs), charitable donations by non-state-owned enterprises (non-SOEs) declined significantly. The charitable donations made by polluting firms in the East of China were significantly lower than those in the Midwest of China.

The possible contributions of this study are two aspects. First, this study enriches the literature on the economic consequences of environmental policies. Combining the “environmental protection fee to tax” policy with corporate social responsibility (CSR) is conducive to a deeper understanding of the effects of environmental protection policy implementation. Second, through the analysis of individual firm differences, this study examines changes in the charitable donation practices of polluting firms before and after the implementation of the “environmental protection fee to tax” policy, which not only provides new evidence regarding the motives behind firm charitable donations but also confirms that the “environmental protection fee to tax” policy has suppressed the hypocrisy of polluting firms. It also provides a reference for the regulatory authorities to improve the legal system and better regulate the charitable donations of polluting firms.

The rest of the paper is organized as follows: the next section documents the literature review, the third section demonstrates institutional change and develops the research hypothesis, the fourth section illustrates the research design, the fifth section presents the empirical results, the sixth section indicates further analysis, and the seventh section provides study conclusions and policy recommendations.

## 2. Institutional Background and Institutional Change

### 2.1. Institutional Background

Over the past thirty years of reform and opening up, China’s economic and social development has made great achievements. Still, the problem of environmental pollution is increasingly prominent, and resource overload and overdraft and environmental pollution are alarming. Nevertheless, the follow-up of environmental protection and unremitting efforts from all walks of life have controlled the rate of environmental degradation, and some conventional pollutants have shown a sustained and steady improvement at the state-controlled cross-sections. However, overall, China is still facing a serious pollution situation. Pollutant emissions are at a very high level and are approaching or even exceeding the environmental capacity. In particular, the frequent outbreaks of severe haze on a large scale in recent years and the obvious emergence of groundwater pollution problems, urban black smelly water bodies, and other problems related to people’s livelihood have made the public’s call for environmental quality improvement increasingly loud. The emergence of this phenomenon is closely related to the unreasonable industrial layout and the sloppy economic development mode. The layout of a large number of heavy chemical firms along rivers, lakes, and rivers poses a serious risk to the quality of the water environment. In addition, various illegal discharges by firms are repeatedly prohibited, becoming an obstacle and bottleneck to green development.

In the face of this serious environmental pollution situation, environmental protection work needs to use a combination of measures and means to play a concerted role in promoting energy saving and emission reduction in firms and industrial restructuring. Therefore, the National Environmental Protection 12th Five-Year Plan proposes accelerating the construction of a resource-saving and environmentally friendly two-type society and taking a series of measures, such as strengthening the construction of the regulatory system, improving environmental and economic policies, and strengthening sectoral coordination to accomplish these goals. From 2016 to 2020, China improved the quality of the environment as the core, solved the outstanding problems in the ecology and environment as the focus, and made every effort to fight the battle to make up for the shortcomings of the ecological environment and protracted war. The 14th Five-Year Plan emphasizes the development of green finance, the formulation of industrial policies conducive to green and low-carbon development, the promotion of cleaner production, the solution of environmental problems in the production process, and the development of the real economy. In such a context, environmental fiscal policy, as an important environmental, and economic instrument, plays the role of a long-term mechanism for adjusting the environmental behavior of firms and promoting the transformation of the development mode.

### 2.2. Institutional Change

On 1 January 2018, China officially implemented the Law of the People’s Republic of China on Environmental Protection Tax promulgated by the Standing Committee of the 12th National People’s Congress on 25 December 2016. This is the first single tax law in the field of ecological civilization construction in China. As a result, 2018 has also become an important turning point in China’s environmental protection system, from the emission fee collection system that took 40 years (1978–2018) to an environmental protection tax system.

The sewage charging system refers to a series of measures by which the state collects sewage charges from dischargers by the relevant standards for paying charges and using legal procedures. In 1978, the State Council’s Leading Group for Environmental Protection proposed “charging pollutants to emission organizations” and formally implemented the charging policy in 1979. Exploration by multiple sectors, a unified and standardized system has been initially formed regarding the content of the sewage charge levy, the levy’s objects, the levy’s procedures, and standards. In 1995, the key industrial sources of pollution to open the levy reached 100%. The levy of sewage charges increases the economic burden of firms emitting pollutants and plays a certain role in eliminating pollution emissions. The levied sewage charges are paid to the treasury and managed through special environmental protection funds after being included in the financial budget. That is the “separation between revenue and expenditure” management model. All the emission fees collected under this model are used for pollution governance. The levy of sewage charges increases revenue and enhances the government’s efforts to combat pollution. 

However, in the process of implementing the sewage charging system, many flaws have been exposed day by day. For example, although the Standing Committee of the National People’s Congress amended and implemented the Air Pollution Prevention and Control Law in 2000 and established a system of charging for total emissions, it failed to solve the dilemma of making ends meet for sewage governance. Local environmental protection sectors and pollutant discharge firms play games, and polluting firms bargain. Local governments place too much emphasis on GDP growth. As a result, the rigidity of charging is weak. Local governments exist to reduce or waive emission fees to reduce the burden on firms and promote investment. These lead to the adoption of an agreement on sewage charges, and the collection rate is not high due to incomplete collection, which cannot achieve the proper policy function of sewage charges. In continuously improving the sewage charge system, the calls for “changing fees to taxes”, “greening the tax system,” and “excluding fees and establishing taxes” are rising day by day. 

In early 2018, the enactment of the environmental protection tax law officially responded to the decision of the Third Plenary Session of the 18th Central Committee to “promote the conversion of environmental protection fees into taxes”. Table 1 shows that the changes to China’s environmental tax law are long overdue and intuitively reflect the central government’s determination for green development and building an ecological China.

The environmental protection tax law follows the guidelines of the object and scope of collecting sewage charges. However, substantial adjustments have been made in how the tax is levied and the tax rate [14]. The aim is to achieve higher emission management and governance standards and motivate firms to improve production efficiency and thus reduce pollution emissions. The tax law also stipulates that the tax sector shall collect the tax according to the law. The environmental protection sector shall assist in the collection. Since then, the levy is no longer an administrative means but has taxation’s rigidity and legal authority. Another important change after the “change of environmental protection fee to tax” is to change the current situation where central and local governments share the pollution discharge fee by 1:9. That is, the central government no longer participates in the share after the “environmental protection fee to tax”, the environmental protection tax as all revenue by local government. This improves the motivation of local governments for environmental regulation, from “turning a blind eye” in the sewage charge period to a more strict and standardized monitoring mode. As a result, the “environmental protection fee to tax” policy reduces the possibility of firms evading their environmental responsibilities through rent seeking and other means from the institutions.

## 3. Literature Review

Previous studies on the motivation of firm charitable donations have mainly included altruistic, self-interest, strategic, and political motivations. Altruistic motivation involves factors such as moral motivation for the public good and regional happiness [15,16]. However, firms are for-profit organizations, and charitable donations often increase operating costs. More studies reveal that firm charitable donation is strategic and can be influenced by multiple factors [17,18,19,20]. Self-interest motives include self-serving decisions by firm management and improper concealment of negative firm news by managers and controlling shareholders [1,21,22]. In addition, firms make charitable donations for strategic and political purposes. For example, ref. [23] finds that peer anchoring motivates firm charitable donations. Ref. [24] finds that firms that join industry associations are more likely to make responsive charitable donations due to their presence in the organization’s context. From a political perspective, firms are incentivized to build and maintain relationships with the government through donations, forming government-business ties, and gaining access to resources and policy benefits. For example, firms can curry favor with the government and the public through charitable donations, thus reducing the suspicion of “original sin” involved in transforming the public to private ownership during the initial acquisition of firm ownership [4]. Ref. [25] finds that private firms’ donations are strategic and can consolidate political connections. For example, politically connected private firms are more willing to donate after an earthquake and receive more tax benefits from bank loans. 

Moreover, prior studies have further verified that private firms have great enthusiasm for establishing good government-business relationships to increase firm value, obtain financing incentives and tax deductions, which reflects that private firms have stronger strategic charitable motives [26,27,28,29]. Furthermore, the tendency and scale of firm charitable donations increase significantly after a change in local government due to changes in affordability, such as access to financing, government subsidies, and investment opportunities [30]. In addition, local officials tend to use the public effect of firm charitable donations to suppress negative news and spread good news when promoted to enhance their “image projects” [31,32].

In addition, since the “environmental protection fee to tax” policy was only formally implemented at the beginning of 2018, existing studies are more concerned with analyzing and comparing environmental protection tax systems between different countries and putting forward evaluations and suggestions for China’s environmental protection tax reform. As a result, there are fewer empirical studies on the economic consequences of implementing the “environmental protection fee to tax” policy for firms. The former study contains mixed reviews of implementing the “environmental protection fee to tax” policy. On the one hand, most existing studies reveal that the “environmental protection fee to tax” is conducive to better use of taxation for active regulation and control, strengthening the monitoring and enforcement of emission behaviors, broadening the collection channels of environmental protection fees, and improving China’s green taxation legal system [33,34,35]. On the other hand, Ref. [36] points out that, due to the current serious problem of information asymmetry in the field of pollution in China, the “environmental protection fee to tax” is likely to lead to an increase in the total cost of taxation and social costs.

Extant research on “environmental protection fee to tax” has focused on firm performance, information disclosure, and green transformation. For example, ref. [8] find that “environmental protection fee to tax” can have a negative impact on the performance of heavily polluting firms, non-SOEs, and firms in economically developed regions such as the eastern region. Ref. [13] argue that there are currently no specific and detailed regulations for firm green information disclosure in China and that there is greater freedom of choice for firm green information disclosure. The “environmental protection fee to tax” policy has increased the cost of taxation for firms and has been subject to stricter monitoring by the authorities and more public attention. Coupled with limited firm resources, funds that would have been used to measure the environment have been squeezed. On the cusp of regulation and public opinion, firms are reluctant to “jackpot” to gain too much attention. As a result, the overall firm green information disclosure is lower. Ref. [37] find that compared to the emission fee system, which has a weaker collection and management constraint and a uniform fee standard, the environmental tax has stronger independence of the collection system, relies on a higher legal system for collection, and has corresponding tax incentives, which can also stimulate technological innovation while increasing the legality pressure on firms and discouraging excessive investment. This “environmental protection fee to tax” policy can promote the green transformation of heavily polluting firms by putting pressure on them to regulate the environment and promote the green growth of firms and the harmonious development of the economy and ecology [38].

The following aspects can be drawn from the above literature on firm charitable donations and the Chinese “environmental protection fee to tax” policy. First, many factors influence firm charitable donations. A review of the literature shows that the vast majority of the literature considers firms to be for-profit organizations whose motivations for making charitable donations are often not purely altruistic but are influenced by various factors such as management and governance within the firm, as well as the public, regulatory authorities and the legal environment outside the firm. Unsurprisingly, studying firm charitable donations remains a hot topic of empirical accounting research. Second, studies on the impact of the “environmental protection fee to tax” policy on firms can be divided into two main categories. The first category is to evaluate and suggest the design of a “fee-to-tax” policy by comparing the environmental taxes of different countries. The other category is the study of the impact of the “fee-to-tax” policy, which involves firms’ performance and technological innovation and transformation. Therefore, existing research has contributed to the CSR and environmental policy field. However, few studies have empirically tested whether implementing the “environmental fee-to-tax” policy affects the charitable donations of polluting firms.

Moreover, from previous studies on charitable giving, most studies have used least squares regression to analyze the linear relationship between a factor and charitable giving, which may lead to problems such as omitted variables. Based on the quasi-natural context of policy implementation, the differences-in-differences approach can well avoid such problems. It not only enriches the literature on charitable giving with a more innovative research approach but also fits the main research content of this study.

Polluting firms have strong negative environmental externalities, and their decisions to make charitable donations differ from those of non-polluting firms. This study attempts to use the implementation of the “environmental protection fee to tax” policy as an exogenous event to investigate the intention of charitable donations by polluting firms and to explore the changes in charitable donations by polluting firms in the context of the policy implementation.

## 4. Theoretical Analysis and Hypothesis Development

China’s ecological protection and environmental governance have gone through nearly 50 years since the United Nations Conference on the Human Environment in June 1972 and have formed an environmental policy system of economic incentives, administrative controls, and public participation. However, once a firm has a major environmental pollution accident or environmental violation, it will damage its reputation and face management penalties. Therefore, to reduce the negative impact on the firm, polluting firms tend to adopt indirect impression management strategies to enhance their image and transfer responsibility through more external communication, such as donations [22,39]. Given this, this study argues that charitable donations are likely to be an important means of polluting firms to divert public attention and evade their environmental responsibilities.

The sociologist Max Weber suggests that the existence of every social system depends on its ability to establish and cultivate a universal belief in the meaning of its existence and that this belief is the legitimacy of its existence. In today’s world of severe environmental pollution, the legitimacy of environmental behavior has become a critical resource for the survival and development of organizations, and due to the real needs of economic development, many firms can not balance environmental protection and profitability. As a result, self-interested firms have minimal motivation to participate in environmental governance [40]. For this reason, firms may use charitable donations to balance firm legitimacy, gain recognition, avoid risks, and obtain governmental recognition and protection of firm legitimacy [41,42,43,44,45,46]. 

In addition, a good relationship between the government and the firm can help firms save resources, achieve firm tax savings, and obtain a relatively relaxed management environment. Charitable donations, as a reflection of firms actively fulfilling their social responsibility and creating firm social value, can promote the formation of implicit bonds between firms and local governments. For this reason, firms are not uncommon to seek rent from the relevant departments through charitable donations for their benefit.

Based on signaling theory, firm charitable giving can send a positive signal to investors. The media focus effect also establishes a good image of corporate social responsibility. In environmental disclosure, when firms emphasize the form rather than the quality of fulfilling their environmental responsibilities, they are more likely to intensify their charitable giving to alleviate external pressures in the face of underinvestment in environmental protection and poor environmental performance [47,48,49].

With the rise of business civilization, reputation is increasingly becoming an important firm resource. A firm reputation is a composite reflection of all of a firm’s past actions and results, which reflect its ability to deliver valuable outputs to a variety of stakeholders. In recent years, the community is increasingly emphasizing the fulfillment of firm environmental responsibility, and firms that cause serious environmental pollution, once reported by the media, are criticized by the community and severely punished by the regulatory authorities, which has a serious negative impact on reputation. On the other hand, firm charitable donations help improve public recognition of the firm, create a good reputation for the firm, and form reputational capital. In contrast, the insurance effect of reputational capital can reduce the cost of administrative control and the risk of social sanctions caused by firm pollution behavior and reduce firm losses when negative news is generated.

The “environmental protection fee to tax” policy is proposed to eliminate heavy-polluting firms, transform light polluting firms, support green firms, and truly put emissions management in place. In China, “tax” and “fee” differ greatly from the collection basis. The collection of environmental protection tax is based on the legal provisions of taxation. The legislative power of taxation is vested in the highest authority. As a result, environmental protection taxation has legal, compulsory, and rigid law enforcement.

In contrast, sewage charges are exercised by local government sectors with greater governmental autonomy. As a result, the collection process reflects a certain degree of arbitrariness. From the collection method, the fee is no longer taken by local administrative means. Instead, the taxpayer shall report to the tax authority, and the tax sector shall collect the tax to ensure the authenticity and legality of the declaration. When tax evasion is found, the tax sector can request the environmental protection sector to assist in auditing the actual pollution emissions of the taxpayer and establish an information-sharing mechanism between the authorities. Regarding tax standards, the environmental tax is set by the state and regulated upward by local governments, ensuring certain flexibility in the collection and the bottom line of the collection [34].

The “environmental protection fee to tax” policy has reduced the possibility of polluting firms evading the responsibility of environmental management through rent seeking and prompted them to shift their attention to the environmental pollution problem instead of spending money on charity to divert public attention from environmental issues and to avoid monitoring by environmental protection sectors. Accordingly, the following hypothesis is proposed in this study.

**H1.**  *Implementing the “environmental protection fee to tax” policy reduces charitable donations from polluting firms*.

## 5. Research Design

### 5.1. Data Source and Sample Selection

The “environmental protection fee to tax” policy is mainly aimed at polluting firms. Non-polluting firms and others are less affected by the policy, which dilutes the effect of policy implementation. Therefore, following ref. [8], this study excludes non-polluting and environmental protection firms. According to the revised Guidelines on Industry Classification of Listed Firms in 2017, all listed firms with primary industry classifications of B, C, and D in Shanghai and Shenzhen A-shares in China from 2015 to 2019 were selected as the initial sample. Shareholders, financial reports, basic information about listed firms, and corporate governance are obtained from the CSMAR database, China’s leading financial data service firm. This study then makes the following deletions: (1) firms in the finance and insurance industries due to their special characteristics; (2) the ST and *ST firms; (3) other samples with missing data. To reduce the effect of variables’ extreme values on this study’s findings, the continuous variables are winsorized at the 2.5% and 97.5% levels. This study ends up with 5359 samples, of which the control group has a sample of 4317, and the treatment group has a sample of 1042.

### 5.2. Research Methodology and Model Setting

On 1 January 2018, the “environmental protection fee to tax” policy was officially implemented. The implementation of policies affects the behavior of firms. However, the behavioral decisions of the firm do not reverse the influence of policy formulation and implementation. Therefore, this study utilizes the implementation of the “environmental protection fee to tax” policy as an exogenous event and uses the DID method to examine the association between the “environmental protection fee to tax” policy and the firm charitable donations. The sample is divided into control and treatment groups and differentiated into two dimensions, individual and time, using 2018 as the time point. Their quadratic differential results are the net effect of the policy. Since not all firms’ tax burden standards have changed during this policy’s implementation, many firms (tax equalization firms) have maintained their original standards. Therefore, following ref. [8], this study takes the change or not in environmental tax burden before and after the policy as the basis for grouping and treats the firms that follow the original standard as the control group and the firms with increased tax burden as the treatment group. The classification criteria for tax equalization and tax burden raising follow ref. [8] and are further determined by checking the tax burden standard set by local governments.

The model is constructed as follows.

(1) $${Donation}_{ij}=\alpha_{0}+\beta_{1}T_{i}+\beta_{2}D_{j}+\beta_{3}T_{i}\times D_{j}+\beta_{4}{Controls}_{ij}+\varepsilon_{ij}$$

where *i* denotes time, and *j* denotes firm. *Donation_ij_* represents firm charitable donations and is the dependent variable. *T_i_* is the time dummy variable. *D_j_* is the grouping dummy variable. *T_i_* × *D_j_* is the interaction term of the net policy effect. *Controls_ij_* is the set of control variables. *α*_0_ is a constant. *β*_1–4_ are the coefficients of the corresponding variables. *ε_ij_* is the overall model error. The binary take of *T_i_* is the difference in the time dimension. *D_j_* is the difference in individual dimensions. Two differentials complete the interaction term *T_i_* × *D_j_*. That is, *β*_3_ reflects the net effect of policy implementation.

### 5.3. Variable Definition and Descriptive Statistics

Following ref. [1,4], this study utilizes the ratio of total charitable donations to total firm assets to measure firm charitable donations, which is used to eliminate the scale effect among firms and multiplies total charitable donations by 1000 to eliminate the grade difference. The missing value of firm charitable donations is assigned to zero by following previous studies [4,30].

The independent variable includes a binary time dummy variable (*T_i_*), a binary grouping dummy variable (*D_j_*), and an interaction term between the two (*T_i_* × *D_j_*). *T_i_* denotes whether the policy is implemented in the year *i*. *T_i_* = 1 for 2018 and subsequent years; otherwise, *T_i_* = 0. *D_j_* represents whether firm *j* is subject to a policy shock resulting in a significant tax burden change. If the firm tax burden is raised, it belongs to the treatment group, *D_j_* = 1. Otherwise, *D_j_* = 0. The interaction term *T_i_* × *D_j_* is the key independent variable. If *j* is the treatment group sample, *T_i_* × *D_j_* = 0 is before policy implementation, and *T_i_* × *D_j_* = 1 is after implementation. If *j* is the control group sample, then *T_i_* × *D_j_* is both 0.

Following previous studies [50,51], this study controls for the potential effects of firm size (Size), gearing (Lev), return on assets (Roa), growth (Growth), cash flow (Lncashflow), overhead (Managepay), and industry and year effects. The variables are defined in Table 2.

## 6. Empirical Results

### 6.1. Descriptive Statistics

Descriptive statistics of the main variables are shown in Table 3. The average value of the firm’s charitable donations is 0.033, with a minimum value of 0 and a maximum value of 0.626, indicating that there is a wide range of charitable donations among different firms. In terms of the nature of ownership, the mean value is 0.343, indicating that the sample size of non-SOEs is larger than that of SOEs. The minimum value of the size of the sample firms is 20.226. The maximum value of the size of the sample firms is 24.116. The average value of the size of the sample firms is 21.882. The difference is not very large.

### 6.2. Baseline Regression Results

DID is performed on the model (1). The results of the baseline regression are shown in Table 4. Columns (2), (3), and (4) control for industry effects, year effects, and industry and year effects, respectively, based on controlling for covariates. The regression results show that the interaction coefficients are always negative and significant at the 5% level, indicating that implementing the “environmental protection fee to tax” policy significantly reduces charitable donations by polluting firms. This fits with the theoretical analysis presented earlier that firms may reduce their charitable giving after tax reform. The empirical results support the research hypothesis. From the control variables: Roa, Size, and Lncashflow regression coefficients are all positive, indicating that the higher the return on assets, the larger the scale, the more cash, and the higher the level of corporate charitable donations, which is consistent with the existing literature conclusions [10]. Based on an exogenous test of “environmental fees to taxes”, this study verifies that the policy reduces the possibility of polluting firms evading their responsibility for environmental management through charitable donations at the institutional level and provides a research reference for the government to judge the “hypocritical” behavior of firms.

### 6.3. Robustness Tests

**Figure 1:**
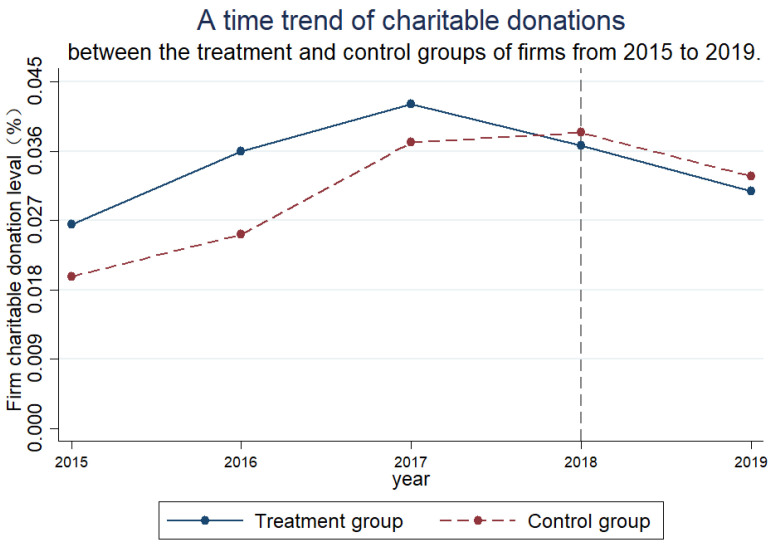
Parallel trend test.

Figure 1 depicts a time trend of charitable donations between the treatment and control groups of firms from 2015 to 2019. Figure 1 shows that before implementing the “environmental protection fee to tax” policy, the charitable donation of firms in the treatment and control groups was the same. The charitable donation of firms in the treatment group was higher than those in the control group. After 2017, the firm charitable donation to the treatment group changed from an overall upward trend to a downward trend. The gap between the two groups of firm charitable donations also decreased significantly, probably because of the adoption of the environmental protection law at the 25th meeting of the 12th NPC Standing Committee on 25 December 2016. Although the policy is not officially in effect, the policy preview has impacted charitable donations by polluting firms. After the implementation of the policy in 2018, the charitable donations in the control group were higher than that in the treatment group, indicating that the implementation of the “environmental protection fee to tax” policy had an eliminating effect on the firm “false charity” and verified the parallel trend hypothesis. After 2018, the firm charitable donation in the tax equalization control group also showed a decreasing trend, suggesting that the firm charitable donation was not entirely affected by the pressure of increased tax burden, i.e., again proving that firm charitable donation is motivated by non-altruistic motives.

This study utilizes a placebo test to exclude the effect of other policies on the empirical results and follows ref. [37,38]. First, assuming that the implementation point of the “environmental protection fee to tax” policy is 2016, this study creates a new time dummy variable. Next, it generates interaction terms between the new time dummy variable and the grouping dummy variable. This study then uses DID. In the study design, the interaction coefficient theoretically failed the significance test. The results of DID are shown in Table 5. The estimated coefficient of the interaction term is positive and does not pass the significance test. The results corroborate the reliability of the above empirical results. Therefore, the results of this study are robust under the placebo test.

To make the research results more robust and follow ref. [4], this study replaces the ratio of total charitable donations to total firm assets with the ratio of total charitable donations to firm operating income. DID is used based on model (1). The results are shown in Table 6. The coefficient of the interaction term is negative and significant at the 5% level. The results are robust.

## 7. Further Analysis

The economic consequences of the “environmental protection fee to tax” policy vary in individual dimensions, mainly in terms of the pollution level of the industry, the nature of the firm’s ownership, and the region where the firm is located. Therefore, to further examine the changes in charitable donations among different types of firms under the implementation of the “environmental protection fee to tax” policy, follow ref. [8], this study groups according to industry pollution differences, ownership differences, and regional differences and still uses DID. The differences in the empirical results of different subgroups suggest that the “environmental protection fee to tax” policy has different effects among different types of firms.

### 7.1. Industry Pollution Difference

The industry to which a firm belongs is critical to its strategic decisions. Polluting firms in different industries have very different pollution emissions due to differences in production processes and product characteristics. Therefore, their donations inevitably influence the industries they belong to [52,53]. Therefore, the “environmental protection fee to tax” negatively impacts the most polluting firms’ charitable donations. There are two reasons. First, the main purpose of implementing the “environmental protection fee to tax” policy is to regulate polluting firms and reduce pollution emissions. Therefore, heavy-polluting firms bear the brunt. With limited resources, high governance costs, and strict environmental taxes, heavily polluting firms are difficult to “take the easy way out” to treat the symptoms but not the root cause [8,54]. Second, heavy-polluting firms are incentivized to lower their sewage charges through rent seeking before the “environmental fees to taxes” change. However, after the “environmental protection fee to tax”, heavy-polluting firms are less likely to seek rent from the government through charitable donations due to the increased cost and difficulty of rent seeking [55].

According to the degree of pollution of the industry to which the firm belongs, this study divides the sample into heavy, moderate, and light pollution groups. The empirical results are shown in Table 7. The interaction coefficients of light and moderate pollution groups were around −0.011, and the coefficients were insignificant. On the other hand, the interaction coefficient of the heavily polluting group is around −0.032 and is significant at the 10% level. The results indicate that charitable donations by heavily polluting firms dropped significantly after implementing the “environmental protection fee to tax” policy. However, charitable donations by light and moderate polluting firms are not significantly affected. This is because moderate- and light-polluting firms have less incentive to reduce environmental controls through charitable donations and are less affected by the “environmental fee-to-tax” policy during the emission fee period. The results also support the study’s main theoretical logic that polluting firms may shift their social responsibility for environmental protection through charitable donations. The empirical results imply that the government should focus on such behavior of heavily polluting firms.

### 7.2. Ownership Difference

On the one hand, SOEs tend to take some significant social responsibility. For example, SOEs tend to set a good example in environmental protection or charity donations. On the other hand, because of political connections, the government makes “charitable apportionments” to SOEs. They even force donations, making charitable donations less optional for SOEs than non-SOEs [30,56,57]. In addition, non-SOEs are subject to higher environmental regulations compared to SOEs. Relatively strict environmental regulatory pressures increase the incentive for non-SOEs to establish good government-business relationships through charitable donations and seek a more relaxed regulatory environment and lower expenditure on emission fees [6,21,24,58,59,60]. However, after the “environmental fees to taxes” policy, the environmental protection system has become more sophisticated and strict. As a result, the cost of evading environmental tax responsibilities has increased. As a result, the marginal benefits of charitable donations by non-SOEs have decreased, lowering the level of charitable donations by non-SOEs.

This study divides samples into SOEs and non-SOEs according to their ownership. The empirical results are shown in Table 8. The coefficient of the interaction term in the SOEs is positive and insignificant. On the other hand, the interaction term coefficient for non-SOEs is around −0.017 and is significant at the 5% level. The results suggest that the charitable donations of non-SOEs decreased significantly after the “environmental protection fee changed to tax” policy. This fits the logical reasoning of the main hypothesis of this study but also confirms that SOEs take on more social responsibility. The results illustrate the need for all parties to pay more attention to the social behavior of non-SOEs to help achieve better all-around green, coordinated, and sustainable development.

### 7.3. Regional Difference

With the advancement of marketization, the concept of “strategic charitable” has gradually taken shape, and firm charitable has become the reciprocal behavior. As a strategic investment, donations can improve a firm’s competitive environment and create a competitive advantage. Firms with stronger marketing capabilities enjoy more dividends from donations [52,61]. The more market-oriented the region, the more willing firms are to build their image, improve their development environment, and reduce the negative impact of firm pollution behavior through charitable donations [57,62,63,64,65]. China has great regional differences and significant regional economic characteristics. The eastern region, by the reform and opening-up policy, has a relatively good economic foundation and a high degree of marketization. The central region shows a steady development, mainly focusing on the agricultural economy, with the second-highest marketization, after the eastern region. The western region has a relatively low degree of marketization and is still at the stage of development driven by the policy of western development and the power of capital. Local governments are more likely to adopt a relatively relaxed regulatory environment to attract investment [10]. The incentive for firms in the western region to rely on donations to seek rents from the local government and thus reduce environmental pollution charges during the emission fee period is relatively weak. There is relatively little for the level of donations to fall after the “environmental protection fee change to tax”.

According to the firms’ regions, this study divides samples into three groups: eastern, central, and western. The empirical results are shown in Table 9. The coefficient of the interaction term is negative for all three sample groups. However, only the coefficient of the interaction term for the sample group in the east is significant at the 10% level. This is because of the high degree of marketization and the number of firms in the eastern region. Compared with firms in the central and western regions, where marketization is relatively low, and the number of competing firms is relatively small, firms in the eastern regions have more incentives to obtain reputational capital and reduce the negative impact of firm environmental pollution through donations. Facing a stricter regulatory environment after the “environmental protection fee to tax” policy, charitable donations from eastern firms have dropped significantly. Comparatively, firms in the Midwest have less incentive to make strategic charitable donations during the emission fee period. Therefore, they are not significantly impacted by environmental policies. The findings support the moderating effect of regional heterogeneity on the relationship between “environmental fees to taxes” and firm charitable donations, suggesting that in the economically developed eastern region, the government needs to pay more attention to whether firm charitable donations are made only to create a good firm image while concealing substantial environmental damage, and provide empirical implications for regulatory authorities to monitor.

### 7.4. Regional Difference Combined Ownership Difference

Based on the empirical results of ownership differences and regional differences, an interesting issue is whether regional difference combined with ownership difference has a more significant effect on the charitable donations of polluting firms. Therefore, this study compares the changes in the charitable donations of non-SOEs in the eastern region and SOEs in the western region before and after implementing the “environmental protection fee to tax” policy. The empirical results are shown in Table 10. Table 10 shows that the interaction term coefficient for eastern non-SOEs is around -0.018 and is significant at the 5% level. On the other hand, the coefficient of western SOEs is negative but not significant. The results are robust.

The western region is less market-oriented. The institutional environment is relatively poor. Concealed information and coordination costs must be considered for polluting firms’ charitable donations to achieve the desired goals. Western regions are mostly large mineral SOEs with less autonomy to choose for charitable donations [66,67]. As a result, non-SOEs in the eastern region have greater incentives to engage in direct and indirect rent seeking through charitable donations before the “environmental fees to taxes” policy. One is to lobby the government to provide the resources firms need and restrict competition. The other is to indirectly influence the government’s or the public’s judgment with a positive image [51,54,68]. Therefore, after implementing the “environmental protection fee to tax” policy, non-state polluters became more difficult in the eastern region to seek rent, making their donations drop significantly. The policy does not significantly affect the western region due to relatively little decline. The results further validate the main hypothesis of this study and the influence of region and ownership on the relationship between “environmental fees to taxes” and charitable donations and provide an implication for the authorities to regulate non-SOEs in the east.

## 8. Conclusions and Policy Recommendations

Environmental pollution has always been a social issue that people are aware of and actively concerned about. As the overall economic development increases, people have higher requirements for environmental protection. Implementing the “environmental protection fee to tax” policy reflects China’s enhanced ecological and environmental protection awareness. It replaces the sewage charge system of the past forty years, providing a new basis for China’s pollution governance. In the face of the “environmental protection fee to tax” policy, polluting firms undoubtedly are affected first. An important issue is how the charitable donations to polluting firms are reflected specifically. This study empirically examines the changes in charitable donations by polluting firms before and after implementing the “environmental protection fee to tax” policy using a DID approach. This study further examines the impact of the “environmental protection fee to tax” policy on polluting firms with different pollution levels, nature of ownership, and regions. The results show that charitable donations from polluting firms dropped significantly after implementing the “environmental protection fee to tax” policy. After implementing the “environmental protection fee to tax” policy, the charitable donations from heavily polluting firms dropped significantly compared to moderately and lightly polluting firms. After implementing the “environmental protection fee to tax” policy, charitable donations from non-SOEs dropped significantly compared to SOEs. After implementing the “environmental protection fee to tax” policy, the charitable donations by firms in the eastern region dropped significantly compared to those in the central and western regions, with private firms in the east being more significantly affected. This study takes the reform of China’s environmental protection tax system as a perspective. It examines firm charitable giving behavior from the perspective of “environmental protection fee to tax”, thus adding and expanding the literature on the economic consequences of the change in the environmental protection tax system and firm charitable giving, as well as having certain policy implications. This study finds that the environmental protection fee to tax change can test the real motives of firm charitable giving and is not purely altruistic, which is an important implication for the government to judge the initial intention of polluting firm charitable giving and to guide firm green development and thus promote the local economy.

The “environmental protection fee to tax” policy aims to promote firms’ green transformation and address environmental problems. Therefore, based on the findings of this study, the following policy recommendations are made.

First, make good use of environmental tax policy and value its environmental importance. The environmental protection tax policy not only raises the tax burden based on sewage charges, which brings more financial flow for pollution governance but also regulates the relationship between the levying sector and the levy recipient by the compulsory nature of laws and regulations so that firms can face their environmental problems and put their resources to good use. Therefore, governments should make good use of the environmental tax policy so that firms can move on to green development and truly achieve the goal of “combating pollution and protecting the environment”.

Second is the implementation of industry-differentiated environmental policies. For industries with different levels of pollution, government sectors should focus. In the early policy implementation stage, the focus should be on monitoring and regulating heavily polluting firms. The regulation of light and moderately polluting firms is at a later stage to effectively improve environmental enforcement and ensure the effectiveness of policy implementation.

Third is strengthening the environmental monitoring of non-SOEs. Compared to SOEs with lower intervention costs, non-SOEs have independent ownership and greater autonomy in taking on CSR, such as environmental protection and public charity. Therefore, non-SOEs are affected much more by implementing the environmental tax than SOEs. Therefore, governments should increase the monitoring of non-SOEs. Furthermore, governments should take the necessary coercive measures against firms that violate the rules of environmental tax collection and urge them to make green renovations and establish proper environmental awareness.

Fourth is implementing environmental policies according to local conditions. The geographical location, development conditions, and management levels of China’s eastern, middle, and western regions are very different. Therefore, the impact of the stricter and higher standard of levy on firms in different regions after the “environmental protection fee to tax” policy is very different. Therefore, governments can give reasonable tax incentives according to the local development conditions. Furthermore, it simultaneously incentivizes their production motivation and reduces firms’ resistance to environmental protection policies.

There are some limitations to this study. First, this study examines the association between the “environmental fee-to-tax” policy and the charitable donations of polluting firms but does not further investigate the effectiveness of the policy in combating pollution. Second, this study uses a DID model and selects the event of the implementation of the “environmental fee to tax” policy as a quasi-natural experiment, which can only examine the short-term effects of the policy because the policy was formally implemented at the beginning of 2018, a relatively short period. However, environmental protection has always been a topic for people. Therefore, research on the long-term effects of environmental policies is the focus of future exploration.

## Figures and Tables

**Table 1:** History of Changes in China’s Environmental Protection Tax Law.

1978	The State Council’s Leading Group on Environmental Protection proposed the idea of a sewage charging system
1979	The Eleventh Session of the Standing Committee of the Fifth National People’s Congress promulgated the Environmental Protection Law of the People’s Republic of China (for trial implementation)
1982	The State Council issued the “Interim Measures for the Collection of Sewage Charges”.
1984	The Ministry of Construction and the Ministry of Finance unified the budget management, budget accounts, and methods of income and expenditure settlement and accounting for sewage charge funds
1985	SEPA’s first national conference on sewage charging put forward the idea of reimbursable use of sewage charge funds
1988	The State Council approved and issued the Interim Measures for the Reimbursable Use of Special Funds for the Treatment of Pollution Sources
1993	The State Planning Commission and the Ministry of Finance jointly issued the “Notice on the Collection of Sewage Sewage Charges”.
2000	The Standing Committee of the National People’s Congress amended and implemented the Air Pollution Prevention and Control Law and established a system of charging for total emissions
January 2003	The State Council issued “regulations on the collection and use of sewage charges”.
February 2003	The former National Development Planning Commission, the Ministry of Finance, and the State Environmental Protection Administration promulgated the Measures for the Administration of Sewage Charges Collection Standards
March 2003	Ministry of Finance and State Environmental Protection Administration announced “Management Measures for the Collection and Use of Sewage Fee Funds”.
July 2003	The official implementation of the three documents issued in the year
2016	The twenty-fifth meeting of the Standing Committee of the twelfth National People’s Congress adopted the Environmental Protection Law
2018	Official implementation of environmental protection “fee to tax” policy

**Table 2:** Definition of variables.

Name	Symbols	Definitions
charitable donations	Donation	(Total charitable donations × 1000)/Total firm assets
time dummy variable	*T*_*i*_	Take 1 after the fee change to tax, and take 0 before the fee change to the tax
grouping dummy variables	*D*_*j*_	The treatment group takes 1, i.e., the province where the firm is located, to raise the tax burden; the control group takes 0, i.e., the province where the firm’s tax burden is equalized.
firm size	Size	Natural logarithm of total firm assets
gearing ratio	Lev	Financial leverage of the firm
return on assets	Roa	(Total profit + finance expenses)/Total assets
Growth	Growth	(Current amount of operating income for the current year − amount for the same period of the previous year)/(Amount of operating income for the same period of the previous year)
Cash flow	Lncashflow	Cash flows from operating activities
Overhead ratio	Managepay	Administrative expenses/operating income
Nature of ownership	Equity	non-SOEs take 0, SOEs take 1

**Table 3:** Descriptive statistics of the main variables.

Variables	Mean	SD.	Median	Min.	Max.
Donation	0.033	0.118	0.000	0.000	0.626
Size	21.882	0.954	21.801	20.226	24.116
Lev	0.370	0.176	0.359	0.079	0.757
Roa	0.041	0.060	0.042	−0.170	0.157
Growth	0.170	0.300	0.122	−0.355	1.167
Equity	0.034	0.180	0.000	0.000	1.000
Lncashflow	18.951	1.314	18.956	16.068	21.725
Managepay	0.092	0.057	0.079	0.018	0.275

**Table 4:** Baseline regression results.

	(1)	(2)	(3)	(4)
	Donation	Donation	Donation	Donation
*T_i_* × *D_j_*	−0.014 ** (−2.035)	−0.014 ** (−2.064)	−0.014 ** (−2.048)	−0.015 ** (−2.077)
*T_i_*	0.011 ** (2.509)	0.018 *** (3.216)	0.011 ** (2.484)	0.018 *** (3.204)
*D_j_*	0.011 ** (2.243)	0.011 ** (2.317)	0.011 ** (2.264)	0.011 ** (2.337)
Constant	−0.214 *** (−4.343)	−0.220 *** (−4.469)	−0.253 *** (−5.105)	−0.258 *** (−5.218)
Control variables	YES	YES	YES	YES
Industry fixed	NO	YES	NO	YES
Year fixed	NO	NO	YES	YES
Observations	5359	5359	5359	5359
Adjusted R^2^	0.017	0.020	0.017	0.020

Note: **and *** indicate significance at the 5% and 1% levels, respectively.

**Table 5:** Placebo test results.

	(1)	(2)	(3)	(4)
	Donation	Donation	Donation	Donation
*T_i_* × *D_j_*	0.004 (0.403)	0.004 (0.403)	0.004 (0.403)	0.004 (0.403)
*T_i_*	0.020 *** (3.117)	0.020 *** (3.117)	0.020 *** (3.117)	0.020 *** (3.117)
*D_j_*	0.009 (1.187)	0.009 (1.187)	0.009 (1.187)	0.009 (1.187)
Constant term	−0.246 *** (−3.464)	−0.246 *** (−3.464)	−0.246 *** (−3.464)	−0.246 *** (−3.464)
Control variables	YES	YES	YES	YES
Industry fixed effects	NO	NO	YES	YES
Time fixed effects	NO	YES	NO	YES
Observations	2692	2692	2692	2692
Adjusted R^2^	0.032	0.032	0.032	0.032

Note: *** indicates significance at the 1% levels, respectively.

**Table 6:** Robustness tests for alternative the dependent variable.

	(1)	(2)	(3)	(4)
	Donation2	Donation2	Donation2	Donation2
*T_i_* × *D_j_*	−0.050 ** (−1.965)	−0.049 ** (−1.967)	−0.050 ** (−1.964)	−0.050 ** (−1.966)
*T_i_*	0.030 (1.272)	0.020 (1.585)	0.029 (1.250)	0.020 (1.566)
*D_j_*	0.016 (0.904)	0.016 (0.917)	0.016 (0.892)	0.016 (0.906)
Control variables	YES	YES	YES	YES
Industry fixed effects	NO	NO	YES	YES
Time fixed effects	NO	YES	NO	YES
Observations	5225	5225	5225	5225
Adjusted R^2^	0.004	0.006	0.008	0.009

Note: ** indicates significance at the 5% levels, respectively.

**Table 7:** The impact of industrial pollution differences.

	Donation		
	(1) Heavy Pollution	(2) Moderate Pollution	(3) Light Pollution
*T_i_* × *D_j_*	−0.032 * (−1.752)	−0.011 (−0.701)	−0.011 (−1.277)
Constant	−0.198 (−1.587)	−0.361 *** (−2.758)	−0.121 ** (−2.231)
Control variables	YES	YES	YES
Industry/time fixed effects	YES	YES	YES
Observations	1016	1444	2858
Adjusted R^2^	0.053	0.026	0.008

Note: *, **, and *** indicate significance at the 10%, 5%, and 1% levels, respectively.

**Table 8:** Results of the effect of differences in the nature of ownership.

	Donation	
	(1) Non–SOEs	(2) SOEs
*T_i_* × *D_j_*	−0.013 * (−1.746)	0.019 (0.798)
Constant	−0.275 *** (−5.364)	−0.052 (−0.586)
Control variables	YES	YES
Industry/time fixed effects	YES	YES
Observations	5184	175
Adjusted R^2^	0.018	0.076

Note: * and *** indicate significance at the 10% and 1% levels, respectively.

**Table 9:** Results of the effect of regional differences.

	Donation		
	(1) Western	(2) Central	(3) Eastern
*T_i_* × *D_j_*	−0.004 (−0.152)	−0.010 (−0.605)	−0.015 * (−1.878)
Constant	−0.138 (−0.731)	−0.146 (−1.409)	−0.296 *** (−4.995)
Control variables	YES	YES	YES
Industry/time fixed effects	YES	YES	YES
Observations	450	856	4053
Adjusted R^2^	0.070	0.024	0.020

Note: *and *** indicate significance at the 10% and 1% levels, respectively.

**Table 10:** Results of the effect of ownership differences combined with regional differences.

	Donation	
	(1) Western SOEs	(2) Eastern Non–SOEs
*T_i_* × *D_j_*	−0.042 (−0.706)	−0.017 ** (−2.033)
Constant	−0.004 (−0.016)	−0.313 *** (−5.135)
Control variables	YES	YES
Industry/time fixed effects	YES	YES
Observations	19	3949
Adjusted R^2^	0.578	0.021

Note: ** and *** indicate significance at the 5% and 1% levels, respectively.

## Data Availability

The data supporting this study’s findings are available from the corresponding author upon reasonable request.
